# 

**Published:** 1891-03

**Authors:** 


### LITERARY.

HEREDITY, HEALTH AND PERSONAL BEAUTY.—By V. Shoemaker, A. M., M. D., F. A. Davis, Pub., Philadelphia. Cloth, $2.50, morocco, $3.00 net. This is a meritorious work of 422 pp., which treats of the human body and the best means of promoting its health and vigor, and preserving its beauty ; with tabulated recipes for compounding many of the more popular cosmetics, tonics, external washes, and household remedies. Under the title of "Man's Spiritual Place in Nature," the author launches into theology in a way which will probably be acceptable to those who have become liberalized in their views of the doctrinal teachings of the older schools, but just what this has to do with the hygienic portions of the volume, we are unable to discern. The chapters which are strictly germane to the subject give a great deal of information, useful to both young and old, such as the construction and care of the skin, finger nails, hair, scalp, teeth, ears, nose, feet, etc., etc. The book will be found to be an excellent guide in the treatment of these members.

We have received from Mr. Lum Smith, publisher of the AGENTS HERALD, Philadelphia, Pa., a copy of the first edition of the Guaranteed Agents' Directory. Each agent's address therein occupies a space ½ x 1½ in., boldly printed on paper gummed on one side. Firms wishing to circularize agents are thus saved considerable expense in advertising and addressing. Book and other agents, should send their permanent addresses and state their business for publication to Mr. Smith. The directory is published every week and will prove invaluable to all agents and to every firm wanting agents. Mr. Smith also wants the permanent addresses of men, women and youths in all parts of the country to distribute circulars at $2.00 per thousand.

THE ANNUAL REPORT OF THE POSTMASTER GENERAL gives a vast deal of information regarding our postal system. The Dead Letter office is of itself a curiosity. The number of letters received there the past year exceeded six and a half million. Of these 319,000 contained valuable inclosures, including $40,000 in money, and over $1,400,000 of negotiable paper, whilst 11,000 contained lottery tickets and nearly 200,000 pictures and papers unfit for circulation.

OUR thanks are due to D. H. Goodwillie, M. D., for the following illustrated treatises, reprinted from the New York Medical Journal:

1. DEAFNESS AS A RESULT OF NASAL AND DENTAL DISEASES.
2. NASAL INTUBATION.

Both of these theses are of value to the profession. Also to the author for—

THE FRANKLINIC INTERRUPTED CURRENT.—A new system of Therapeutic Electricity; by Wm. James Morton, M. D., Professor of Diseases of the Mind and Nervous System, etc., etc., New York, freely illustrated.

MODERN COOK BOOK, ILLUSTRATED, Mast, Crowell & Kirkpatrick, Springfield, Ohio. This is a duo. of 320 pp., containing more than a thousand recipes and practical suggestions to housekeepers. Compiled by Mrs. T. J. Kirkpatrick, and completely indexed. We know of no better book of the kind extant. Read in our advertising columns the extraordinary offer of this book, with HALL'S JOURNAL OF HEALTH, and THE LADIES HOME COMPANION, for twelve months, on receipt of *One dollar* at this office. Either one of them is worth the money.

HERBA VITA.—Physicians are using this remedy with their patients, As either a *tonic* or a *laxative it is without a peer*. See advertisement and send 10 cents for sample.

